# State earned income tax credits and general health indicators: A quasi‐experimental national study 1993‐2016

**DOI:** 10.1111/1475-6773.13307

**Published:** 2020-07-08

**Authors:** Erin R. Morgan, Heather D. Hill, Stephen J. Mooney, Frederick P. Rivara, Ali Rowhani‐Rahbar

**Affiliations:** ^1^ Harborview Injury Prevention and Research Center University of Washington Seattle Washington; ^2^ Department of Epidemiology School of Public Health University of Washington Seattle Washington; ^3^ Daniel J. Evans School of Public Policy and Governance University of Washington Seattle Washington; ^4^ Department of Pediatrics School of Medicine University of Washington Seattle Washington

**Keywords:** health, mental health, policy, poverty, tax policy

## Abstract

**Objective:**

To assess the relationship between the presence and generosity of state‐level Earned Income Tax Credits (EITC) and multiple self‐reported measures of general health.

**Data Sources:**

Data on state‐level tax credits and covariates were obtained from the National Bureau of Economic Research and University of Kentucky Center for Poverty Research, respectively. These data were merged with Behavioral Risk Factor Surveillance System survey records from 1993‐2016.

**Study Design:**

Using difference‐in‐differences approaches and survey‐weighted Poisson regression that accounted for clustering of observations and included state and year fixed‐effects, we assessed relationships between EITC and self‐reported overall health, frequent mental distress, and frequent poor physical health in the prior 30 days. Covariates included state minimum wage, state GDP, and adoption of Medicaid expansion. Sensitivity analyses revealed that parallel trends were plausible; there were no significant lead and lag effects.

**Data Extraction Methods:**

Analyses were restricted to respondents with no more than a high school diploma or equivalent because less‐educated adults are more likely to be low‐wage earners and therefore qualify for EITC.

**Principal Findings:**

Among adults with no education beyond high school (*n* = 2 884 790), each additional 10‐percentage‐point increase in the generosity of state EITC—relative to the federal credit—was associated with fewer reports of frequent mental distress (−97.3 per 100 000; 95% CI: −237.2, 42.6) and frequent poor physical health (−149.6 per 100 000; 95% CI: −284.4, −14.9). When restricted to individuals interviewed during the three months when tax rebates are commonly disbursed, the magnitude of the association between EITC and prevalence of reported frequent mental distress was greater (−329.7 per 100 000; 95% CI: −636.0, −23.5).

**Conclusions:**

The generosity of state EITC policies is positively associated with significant reductions in frequent mental distress and poor physical health, especially during months when the credit is received. Interventions to reduce poverty may positively impact health by reducing material hardship and stress.

**What This Study Adds**

- Increased generosity of state Earned Income Tax Credits benefits statewide physical and mental health of all adults with no more than a high school education—benefits are not limited to single mothers
- Positive impacts of state Earned Income Tax Credits are most pronounced in early spring when the benefits are received especially the effect on frequent mental distress
- Social policies such as the state Earned Income Tax Credits influence people beyond economic outcomes and maybe another way to improve health and reduce disparities

**What We Know**

- Earned Income Tax Credit is known to promote employment and is largest anti‐poverty program for working‐age Americans
- Earned Income Tax Credit has previously been found to improve maternal and child health outcomes such as reduced complications in complications healthier birth weight and better child development outcomes

[Correction added on 27 July 2020, after first online publication: the bulleted items in ‘What This Study Adds’ and ‘What We Know’ sections have been corrected.]

## 1 INTRODUCTION

Many risk factors for suboptimal health share poverty as an upstream determinant.^1^, ^2^ Financial precarity is a contributing factor for mental and physical illness.^1^, ^3^, ^4^, ^5^ Income and employment strongly predict health outcomes, especially among those living at or below the poverty line.^1^, ^6^, ^7^ In 2000, there were an estimated 291 000 deaths attributable to poverty and income inequality in the United States (US).^8^ Some of these impacts are so substantial that they contribute to intergenerational health disparities in mental and physical health.^1^, ^9^

Poverty can compromise health via different pathways.^4^ Insufficient income is associated with food insecurity, lack of health insurance, and exposure to environmental hazards (eg, lead paint and air pollution).^10^, ^11^, ^12^ Poverty has potentially long‐lasting psychological and physiological affects through stress processes. Low‐income families are more likely to live in under‐resourced neighborhoods with insufficient infrastructure and high crime rates; they are also more likely to depend on unpredictable employment.^13^ These stressors not only contribute to higher rates of anxiety and depression among low‐income adults^4^, ^14^ but also to overloading and fundamentally altering the body's stress response system causing long‐term damage to one's health.^15^, ^16^

The co‐occurrence of mental and physical health concerns may be caused by shared upstream determinants like poverty. This may be partially explained by the physiological manifestation of stress, or allostatic load, causing long‐term damage to one's health.^16^ In addition, poor physical and mental health have a bidirectional relationship with poverty if they disrupt work, which can create feedback loops perpetuating these problems.^17^, ^18^ Given the important and lasting impact of poverty on health, policies that reduce poverty may produce large improvements to population health across generations.^19^

The federal Earned Income Tax Credit (EITC) was introduced in 1975 as a federal tax credit to low‐earning working families.^20^ Currently, the EITC is the largest anti‐poverty program for working‐aged adults in the US^21^ The credit received by EITC beneficiaries as a tax refund is based on pretax earnings, marital status, and the number of children in the household.^22^ It is “refundable,” meaning that tax filers can receive the credit even if they do not have tax liability. In 2018, approximately 25 million beneficiaries received about $63 billion from the federal program.^23^ Since 1975, many states have enacted their own EITC policies as a supplement to the federal credit and, as of 2017, 29 states and the District of Columbia had their own EITC.^22^ State EITCs were implemented in different years and vary in their generosity between states and over time. This variation creates the opportunity to examine the impacts of EITC policies on health at the state population level using a quasi‐experimental design.

Much of prior literature on the intersection of EITC and health has focused on maternal and child health, reflecting the primary target population of the policy.^24^, ^25^, ^26^ EITC may reduce complications in pregnancy, adverse birth outcomes, and unfavorable child development at individual population levels.^24^, ^25^, ^26^, ^27^, ^28^, ^29^ The consistency of findings across study designs and outcomes supports a causal impact of EITC on maternal and child health. However, recent studies of EITC and multiple measures of short‐term health outcomes for children found few associations.^27^

The relationship between EITC and adult health more broadly has not been well studied.^30^ Authors of a 2013 systematic review reported that the evidence for the impact of EITC policy on measures of overall adult health and well‐being was limited for some outcomes (eg, self‐reported health, mental health/psychological distress, and frequency of poor physical health days) and missing entirely for others (eg, diagnosed mental illness and alcohol consumption). A study of EITC and general health focused on federal policy expansions occurring in the 1990s and spanned 1993‐2001.^31^ Though this study found a relationship between EITC and reduced blood pressure, fewer poor mental health days, and fewer poor physical health days, the study population consisted only of women with children and a high school education or less—which is not uncommon in the extant literature on health and EITC. While women with children in the home are more likely to receive EITC benefits, this inclusion criterion does not capture fathers providing care for their children or individuals without children who may benefit from the tax credits and also substantially limits the size of the study population.

Since 1993, 19 states and the District of Columbia have created refundable EITCs or made existing EITCs refundable.^5^, ^32^, ^33^ Increasing the availability and generosity of the EITC (eg, via state supplements) could likely provide several health benefits for low‐income single mothers.^34^ We sought to provide a contemporary assessment of the effect of state‐level EITC policies on general indicators of physical and mental health which incorporated these recent changes in policy. Identifying policies that improve well‐being could substantially contribute to public health efforts.

We assessed the prevalence of reported poor overall health, frequent mental distress, and frequent poor physical health in relation to state EITC among US adults with no postsecondary education. The knowledge generated by this investigation can inform policymakers of the health benefits of EITC implementation and generosity, which go above and beyond the policy's direct outcomes of employment, income, and reduced poverty.

## 2 METHODS

### 2.1 Study design and population

We utilized cross‐state variation in the existence and generosity of state EITC and compared repeated cross‐sections from the Behavioral Risk Factor Surveillance System (BRFSS) to assess general health outcomes annually in the United States from 1993 to 2016. Conducted by the Centers for Disease Control and Prevention (CDC), BRFSS uses random digit dialing to survey noninstitutionalized adults at least 18 years of age about health‐related risk behaviors, chronic health conditions, and use of preventive services. In the earliest years of BRFSS, some states sporadically failed to participate or submit survey responses to CDC resulting in a loss of three‐state‐years of data: Wyoming in 1993, Pennsylvania in 1994, and Washington DC in 1995.

We restricted our analysis to adults who had no more than a high school education or GED equivalent, a population of workers who are most likely to be affected by state EITCs. Annual earnings would be the most direct measure of eligibility for the EITC, but it is problematic to restrict the sample by an outcome directly affected by the policy and plausibly on the path between the policy and health. Education is a reasonable proxy for EITC eligibility, while also not being affected directly by the policy.^35^ Adults without postsecondary education frequently have lower earnings and are therefore most likely to be eligible for and benefit from EITC policy.^36^, ^37^ After excluding respondents with postsecondary education, the study period included 1272 state‐years of data (DC being treated as a state) with 2 884 790 respondents with high school education or less (characteristics of individual respondents can be found in Appendix S1); a subset were the 743 174 observations included in the analysis of outcomes collected during tax season in which rebates are usually sent (February‐April of each year).^27^, ^38^

### 2.2 Outcomes

The outcomes of interest included the following: (a) suboptimal overall health, (b) frequent mental distress (FMD), and (c) frequent poor physical health (FPPH). Participants were first asked to rate their overall health on a Likert scale from 1 to 5, with 1 indicating excellent health and 5 indicating poor health (the exact wording of the question and labeling of the scale can be found in Appendix S2). Respondents ranking health as “fair” or “poor” were considered to have suboptimal overall health. FMD and FPPH were measured via the “Healthy Days” component of the survey. FMD was assessed through the question, “Now thinking about your mental health, which includes stress, depression, and problems with emotions, for how many days during the past 30 days was your mental health not good?” As has been suggested by prior work by CDC, individuals reporting 14 or more days of poor mental health were considered to have FMD.^39^ Similarly, participants were asked, “Now thinking about your physical health, which includes physical illness and injury, for how many days during the past 30 days was your physical health not good?” A cut‐off of 14 or more days of health being not good was also used for physical health.

Though they are nonspecific, broad indicators of mental and physical health are readily available and informative in surveillance and research.^40^ Public Health Practice agencies ranging from the local to national levels frequently use these indicators in assessing the overall health of the population and in planning.^41^, ^42^, ^43^ The measures have been used by health plans and researchers as a measure of overall physical and mental health and been found to appropriately map on to chronic conditions.^44^, ^45^

### 2.3 Policy exposure

**Figure 1:**
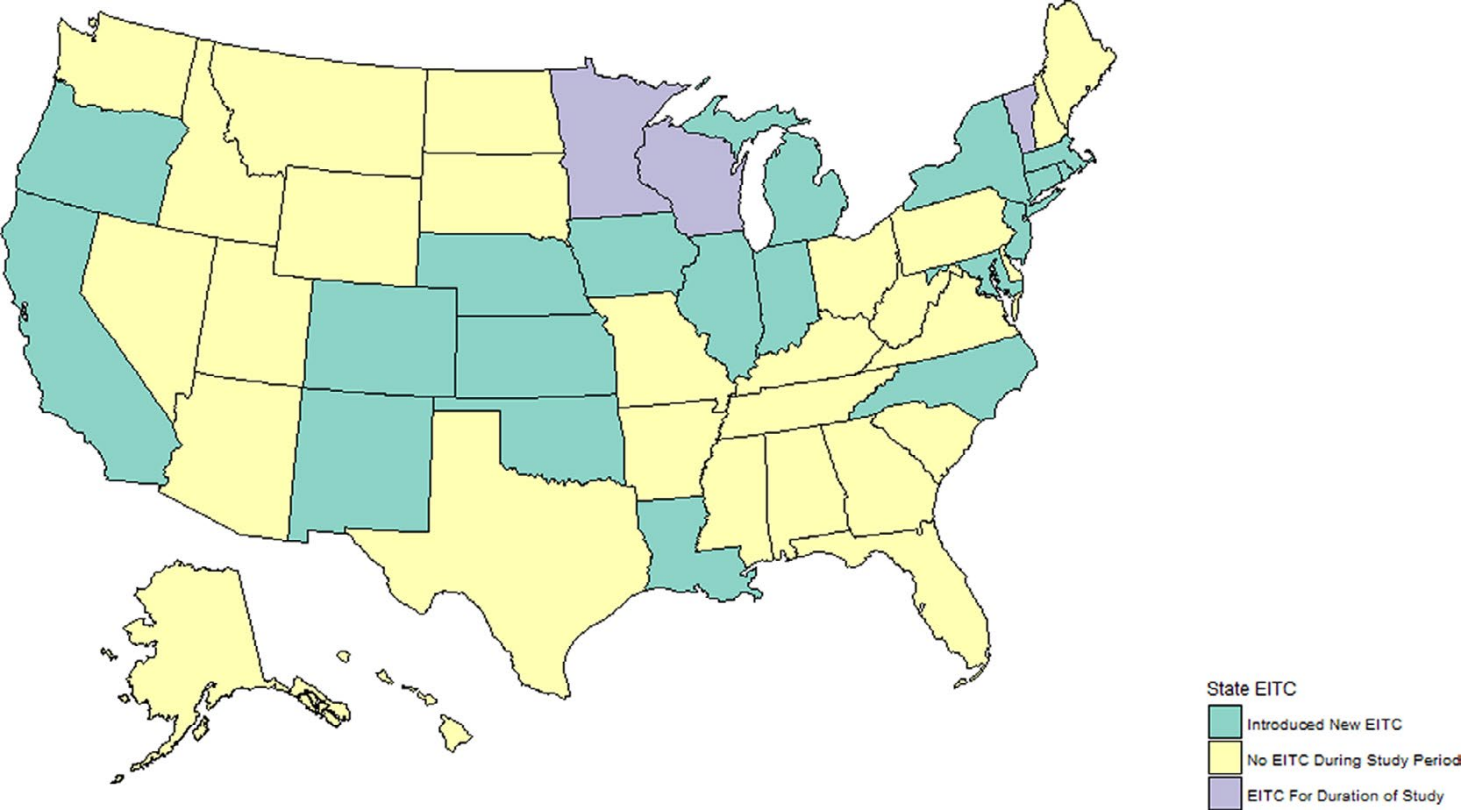
Presence of refundable state earned income tax credit 1993‐2016. [Color figure can be viewed at wileyonlinelibrary.com] *Note: *Green: New Refundable State EITC Introduced/Existing State EITC Made Refundable. Yellow: No Refundable State EITC During Study Period. Purple: Refundable State EITC Available for Duration of Study Period. EITC, Earned Income Tax Credit

The primary exposure was the existence and generosity of a state EITC. State EITC status is presented in Figure 1. These data were obtained from the National Bureau of Economic Research.^33^ In most states, the benefits are set as a percentage of the federal benefit. In our analysis, states with nonrefundable EITCs—where the value of the credit cannot exceed the recipient's tax liability—were considered to not have an EITC because individuals who qualify for the EITC benefits tend to have limited tax liability and thus would not receive any substantial rebate.

Three states are exceptions to the usual pattern of state EITC. The Wisconsin EITC follows the structure of the federal EITC, but its value varies with the number of dependents. In these analyses, the EITC value assigned to Wisconsin reflects the amount received by households with two dependents; this was 25 percent of the federal credit in 1993, 16 percent in 1994 and 1995, 14 percent from 1996 to 2010, and 11 percent from 2011 onward. Minnesota's working family credit is similar to the federal EITC but is more dependent on household income; we used the mean state benefit received by households in Minnesota that qualified for the benefit, which was 15 percent of the federal credit from 1993 to 1997, 25 percent in 1998 and 1999, and 33 percent of the federal credit from 2000 to 2016. The structure of the California EITC differs from the federal benefit in that the value of the credit increases more sharply at a lower income and begins to phase out at a lower income‐level than the federal. California did not introduce the state EITC until 2015, and benefits were not received until 2016 meaning the difference in structure had minimal impact on the analysis; consequently, we did not account for differences in the EITC structure.

### 2.4 Covariates

**Figure 2:**
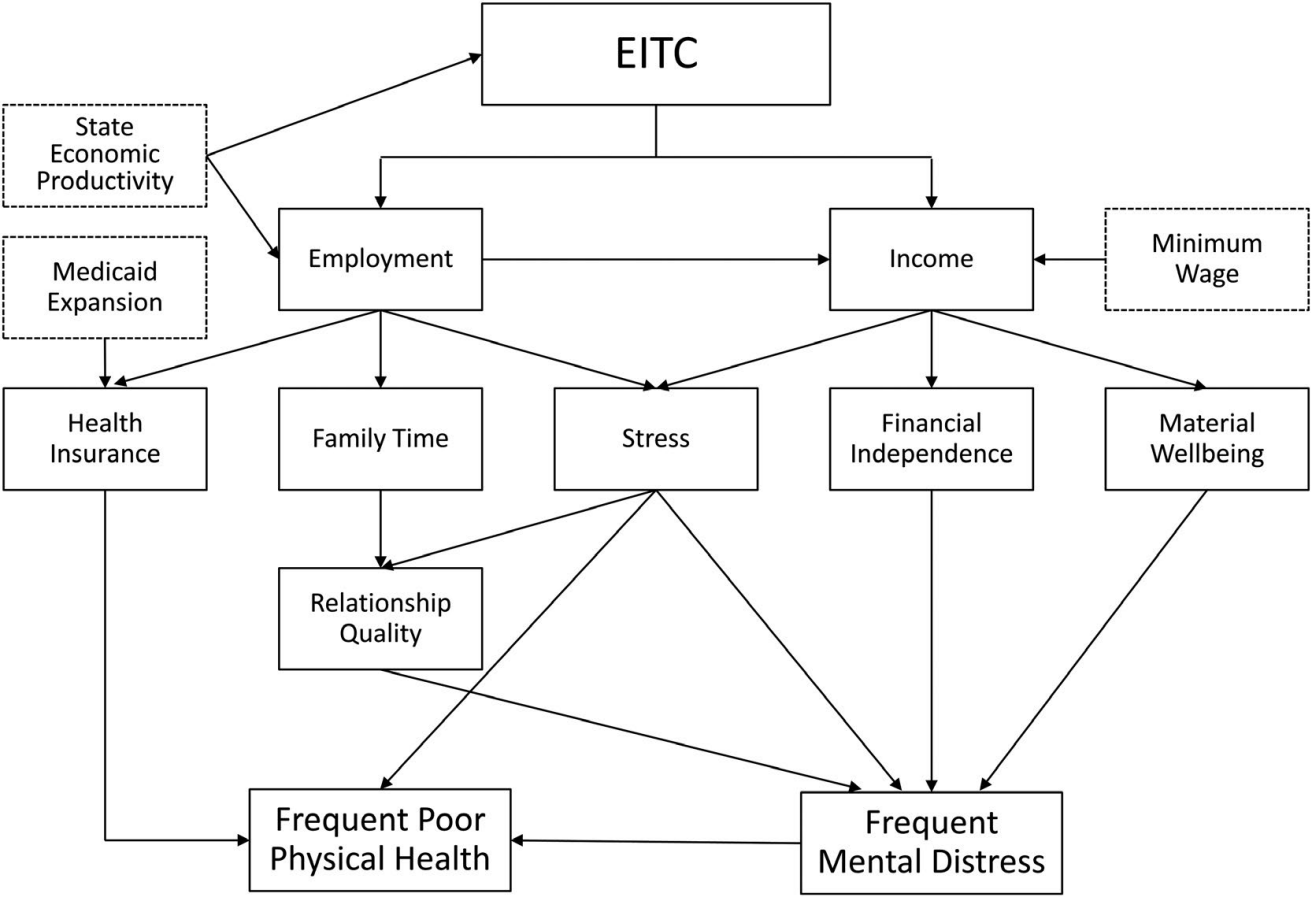
Conceptual model for impact of earned income tax credit on heath outcomes. EITC, Earned Income Tax Credit

We controlled for a set of time‐varying state‐level covariates that could have changed over the study period differentially by state and that are plausibly related to the outcomes of interest. Time‐varying covariates included in the model were state minimum wage (in 2010 inflation‐adjusted dollars) and state GDP (in 2010 inflation‐adjusted dollars). We elected to include minimum wage as a measure of a low‐wage worker's earning potential. The state GDP served as an indicator of the overall economic productivity in a state; after including state and year fixed‐effects, any large changes in the state GDP represent changes in the fiscal landscape that are unique to that state. These financial covariates were obtained from the University of Kentucky Center for Poverty Research (UKCPR) database.^5^ We additionally included an indicator for adoption of Medicaid expansion. After expansion of Medicaid, many low‐wage workers who may have been previously uninsured or under‐insured would have increased access to health care, potentially impacting physical and mental health. Indicators of Medicaid expansion were adapted from data provided by the Henry J. Kaiser Foundation.^46^ These covariates were selected a priori based on existent literature and our conceptual model (Figure 2). Covariates were available for all state‐years of the study period.

While they are important factors in health, we intentionally excluded direct measures of employment and insurance from our models. The EITC can operate through employment which would then potentially impact insurance status. Due to the possible endogeneity of these values and the potential for them to act as mediators on the causal pathway, we did not include them in the models.

### 2.5 Statistical analysis

This analysis used a difference‐in‐differences approach, comparing the change in prevalence of each health outcome across states with more and less generous EITC benefits as the benefits changed. In fully adjusted models, we included state and year fixed‐effects and time‐varying state‐level covariates hypothesized to be related to both EITC generosity and health. We used survey‐weighted Poisson regression and the margins command to calculate prevalence differences (PDs) and 95% confidence intervals (CIs). These models took the following form:

$$\log\left(Y_{\mathrm{itj}}\right)=\alpha +\beta_{1}{\text{EITC}}_{\mathrm{tj}}+\beta_{2}\sum X_{\mathrm{tj}}+\beta_{3}j+\beta_{4}t+\varepsilon_{\mathrm{itj}},$$

where *i* indexed individuals, *t* years, and *j* states. *Y_itj_* was one of three general health outcome measures. *EITC_tj_* denoted a continuous variable, for which each one‐unit increase represents a 10‐percentage‐point increase in the state refundable EITC (as a percentage of the Federal EITC). *X_tj_* was a vector of state‐year covariates and *j* and *t* were state and year fixed‐effects, respectively.^47^ Error terms in analyses were clustered by state. The complex survey weighting method used in the BRFSS took clustering into consideration during calculations. We additionally conducted sensitivity analyses using weighted linear regression.

For difference‐in‐differences models to produce unbiased estimates of the causal effect of the policy, the parallel trends assumption must be plausible. The assumption is that the trends in the outcomes being studied would be parallel in state with and without an EITC before the implementation of the policy and continue the same trend if not for the implementation of the policy. It is not possible to observe the counterfactual after implementation, but it is conventional to examine parallel trends prior to the intervention as evidence of the plausibility of the assumption. We assessed parallel trend assumptions visually (Appendix S3) and quantitatively via two different methods. First, the prevalence of the outcome in each state over time until the point at which EITC was introduced was visually compared between states that did and did not introduce a refundable EITC during the study period. To address this issue quantitatively, we included an interaction term between continuous calendar year and an indicator of the eventual introduction of a refundable state EITC in a Poisson model, in addition to fixed‐effects for ever introducing an EITC, year, minimum wage in 2010 inflation‐adjusted dollars, gross state product in 2010 inflation‐adjusted dollars, and the adaption of Medicaid expansion for all data points prior to EITC introduction—number of state‐years thus varied by state. Additionally, we conducted analyses including model terms for 3‐year lead, 2‐year lead, and 1‐year lead of the EITC exposure.

We anticipate that effects of EITC on mental distress and physical health would operate through stress reduction and thus would take effect shortly after receipt of cash. It follows that if the state EITC impacts mental distress, this effect should be stronger in the months immediately following receipt. To consider the shorter‐term effects of EITC, additional analyses restricted BRFSS responses to interviews conducted during the tax season months of February‐April.^20^, ^38^ This period has been used in prior analyses of the EITC to assess the policy's immediate impact.^27^, ^28^ An additional sensitivity analysis restricted to female survey respondents as single mothers are most likely to receive EITC benefits. All analyses were completed with Stata release 14.2 (StataCorp LP). This project was approved by the University of Washington Institutional Review Board.

## 3 RESULTS

From 1993 to 2016, 28 states did not have an EITC and 3 states had an EITC for the entire period, while 19 states and the District of Columbia implemented a refundable EITC or transitioned from a nonrefundable EITC to a refundable credit (Table 1). During the study period, among adults with no more than a high school education, the nationwide prevalence of suboptimal overall health was 24 020 per 100 000 (95% CI: 23 920‐24 120 per 100 000), of FMD was 12 654 per 100 000 (95% CI: 12 574‐12 734 per 100 000), and of FPPH was 14 221 per 100 000 (95% CI: 14 141‐14 300 per 100 000). During the 24‐year study period, the prevalence of suboptimal health, FMD, and FPPH increased in every state.

**Table 1:** Social and economic characteristics of surveyed states by presence of state EITC, 1993‐2016

	No EITC during study period (*n* = 28)	EITC for full study period (*n* = 3)	Introduction of EITC during study period (*n* = 20)
Median adult population (2000)	2 036 105	3 643 977	3 237 230
Median adult population (2010)	2 213 555	4 027 516	3 623 968
Mean minimum wage during study period in 2010 inflation‐adjusted USD (SD)	$6.60 ($0.89)	$6.96 ($0.77)	$6.86 ($1.09)
Mean State GDP during study period in millions, 2010 inflation‐adjusted USD (SD)	$194K ($240K)	$156K ($108K)	$327K ($400K)
% expanding Medicaid in 2014	35.7%	66.7%	70.0%
Mean prevalence of suboptimal overall health at baseline per 100 000 (SD)	20 018 (5098.5)	15 813.3 (1309.9)	18 546 (3489.5)
Mean prevalence of frequent mental distress at baseline per 100 000 (SD)	9499.3 (1967.2)	8956.7 (2357.2)	8722.5 (2258.7)
Mean prevalence of frequent poor physical health at baseline per 100 000 (SD)	11 689.3 (2240.6)	10 540.0 (928.0)	10 641.5 (2114.0)

Note

Unless otherwise indicated means for the entire study period are displayed—these variables were time‐varying in analytic models

Abbreviations: EITC: Earned Income Tax Credit; SD: standard deviation; USD: United States dollars.

Our main analyses estimating health outcomes as a function of state EITC generosity (Table 2), controlling for state and year fixed‐effects, found that a 10‐percentage‐point higher state EITC, relative to the federal credit, was associated with a decrease in prevalence of both FMD (−260.2 per 100 000; 95% CI: −389.1, −131.2) and FPPH (−211.0 per 100 000; 95% CI: −336.8, −85.3). There was no indication of any relationship between EITC and suboptimal overall health. After adjusting for time‐variant state policies (state minimum wage, state domestic product, and Medicaid expansion), the negative association remained statistically significant for FPPH (−149.6 per 100 000; 95% CI: −284.4, −14.9). While the estimate for FMD was no longer significant after the inclusion of additional covariates, findings were suggestive of a negative relationship (−97.03 per 100 000; 95% CI: −237.2, 42.6). The results for suboptimal overall health changed only marginally from crude estimates and remained nonsignificant. Findings did not differ substantially in sensitivity analyses using a weighted linear regression (Appendix S4).

**Table 2:** Prevalence differences in prevalence of poor health reported on BRFSS per 100 000 population with maximum educational attainment of high school diploma, 1993‐2016

	Crude PD	Adjusted PD^a^
Overall Suboptimal Health	19.3 (−126.1, 164.8)	31.3 (−123.3, 185.9)
FMD	*−260.2 (−389.1, −131.2)*	−97.3 (−237.2, 42.6)
FPPH	*−211.0 (−336.8, −85.3)*	*−149.6(−284.4, −14.9)*

Abbreviations: BRFSS, Behavioral Risk Factor Surveillance System; FMD, Frequent Mental Distress; FPPH, Frequent Poor Physical Health; PD, Prevalence Difference.

^a^ Adjusted for state GDP, state minimum wage, and adoption of Medicaid expansion

Italic values are indicates statistical significance at the p=0.05 level.

When we further focused on the period in early spring (Table 3), when credits were most likely received, the magnitude of the of EITC effect increased for all outcomes. In crude models, FPPH was significantly associated with state EITC (−310.5 per 100 000; 95% CI: −589.8, −31.1) as was FMD (−453.2 per 100 000; 95% CI: −738.7, 167.6). There was a suggestion of a relationship between EITC and improved overall health outcomes; the magnitude of the association was stronger than the year‐round estimate (−158.9 per 100 000; 95% CI: −478.6‐160.8). After adjusting for the same state‐level covariates above, the relationship between EITC and FMD remained statistically significant (−329.7 per 100 000; 95% CI: −636.0, −23.5). The association with FPPH became marginally nonsignificant when including the time‐varying covariates (−267.7 per 100 000; 95% CI: −562.6‐27.1). The results from the adjusted model suggest that suboptimal overall health may be somewhat indicative of improved health, though this was not statistically significant (−128.8 per 100 000; −468.5‐210.9). Additionally, findings were more pronounced when restricting to female respondents to the survey (Appendix S5).

**Table 3:** Prevalence differences in prevalence of poor health reported on BRFSS during February, March, and April per 100 000 population with maximum educational attainment of high school diploma, 1993‐2016

	Crude PD	Adjusted PD^a^
Overall Suboptimal Health	−158.9 (−478.6, 160.8)	−128.8 (−468.5, 210.9)
FMD	−*453.2 (−738.7, −167.6)*	−*329.7 (−636.0, −23.5)*
FPPH	−*310.5 (−589.8, −31.1)*	−267.7 (−562.6, 27.1)

Abbreviations: BRFSS, Behavioral Risk Factor Surveillance System; FMD, Frequent Mental Distress; FPPH, Frequent Poor Physical Health; PD, Prevalence Difference.

^a^ Adjusted for state GDP, state minimum wage, and adoption of Medicaid expansion

Italic values are indicates statistical significance at the p=0.05 level.

When testing parallel trends assumptions, we found no indication of an interaction between future EITC status and calendar year on reported suboptimal overall health (*P* = .073), FMD (*P* = .980), or FPPH (*P* = .670) suggesting that the parallel trends assumption was plausible. None of the lead terms were statistically significantly associated with decreased reports of poor health suggesting that differences in outcomes following the introduction of EITC are not attributable to existing trends.

## 4 DISCUSSION

To our knowledge, this is the largest state‐representative study to assess the impact of EITC on the prevalence of self‐reported health measures. We found that among those with no postsecondary education, the prevalence of frequent mental distress and frequent poor physical health notably decreased with increased generosity of refundable state EITC. These differences in poor health were more pronounced during early spring when EITC benefits are received. They were also larger for women than for men. There was no association between state EITCs and self‐reported suboptimal or poor overall health.

Many prior studies of state EITCs have focused on maternal and child health. Those that have focused on broader health outcomes have been frequently restricted to single mothers.^30^, ^31^ Our results build upon existing literature by assessing the relationship between state EITC and general measures of health among all members of the population likely to be affected (ie, those with lower levels of educational attainment), while still including men, married women, or women without children in the sample. Our analyses found that higher values of state EITC were associated with a lower prevalence of reported poor mental and physical health, which is congruent with prior research that focused exclusively on women with children.^31^ It is reassuring that our study which covered a longer time period—thus including additional changes in EITC—and used a broader study population, results were consistent with prior studies.

While our work was focused on a broader population than much of the prior literature, the research assessing maternal and child health in relation to EITC is helpful in contextualizing these findings. The positive impact of EITC on health becomes more pronounced during the February–April period when eligible individuals tend to receive their EITC benefits.^27^, ^48^ Our findings add to the growing body of literature indicating health benefits immediately following the dispersal of state EITC funds^27^ and are consistent with the notion that the induction period, or time between exposure and observable manifestation of the outcome, is shorter for financial stress and mental health than for the physical health impacts of poverty.

Based on the evidence of relationships between poverty and mental illness, prior work has also investigated EITC in relation to suicide. These analyses found that increased values of refundable state EITCs were associated with decreased suicide rates.^49^, ^50^ These findings were particularly pronounced among women—a group who is more likely to benefit from EITC.^50^ While authors of both papers hypothesized mechanisms by which EITC may decrease risk for suicide, neither study was able to provide evidence. Though the available data precluded formal mediation analyses, our findings that state EITCs are associated with a lower prevalence of frequent mental distress and frequent poor physical health may help explain the relationship between EITC and decreased rate of suicide. Future research using individual longitudinal data may be able to examine causal mechanisms more closely.

In 2013, expenses for mental health and substance use totaled approximately $187.8 billion nationwide and the expenditure for all conditions in the same year was $2.1 trillion in the US.^51^ Though evidence suggests that EITC does not decrease adult health care spending in the short‐term, the long‐term impact of EITC on health‐related expenses is unknown.^28^ There are many programs designed to reduce the burden on the health care system by encouraging healthier lifestyles and reducing the prevalence of disease. While these health‐specific policies and interventions improve quality of life for many, it is important to consider other types of policies, such as EITC, which may also be beneficial in improving health outcomes.

EITC may improve health in multiple ways. The EITC’s cash transfer mechanism is one way that this policy can improve health. The influx of money may allow recipients the immediate opportunity to purchase previously unaffordable medications or seek medical care. Similarly, the sudden increase in resources directly following receipt of the credit can ease stress caused by pecuniary disadvantage; this mechanism is particularly supported by reductions in frequent mental distress observed during February‐April interviews. Other possible immediate behavior changes (eg, changes in dietary patterns, engagement with physical leisure time activities, or access to smoking cessation aids) in addition to the lessened burden of strained financial resources could impact health in the longer‐term.

In addition to the transfer of funds, EITC has been successful as an anti‐poverty measure by further incentivizing employment and decreasing unemployment rates.^20^ Unemployment itself has long been known to worsen mental health, so it is plausible that a policy which increases employment rates decreases frequent mental distress.^4^, ^52^ Additionally, by increasing employment rates, many individuals impacted by EITC are likely to see growth in household income that is reflective of increases in earned wages, in addition to the benefits received through the tax return. This improved financial stability could potentially decrease stress in ways not limited to the early spring period, which is supported by our findings of decreased reports of frequent mental distress and frequent poor physical health in interviews occurring at all times of the year.

Finally, though the additional increase in funds through either cash transfer or increased employment may allow for further contact with the health care system, it is also important to consider the role of insurance. In the US, insurance status is tied to employment. While many part‐time and low‐wage workers may not be eligible for employer‐sponsored insurance, some EITC beneficiaries may qualify. Increased insurance coverage allows individuals to seek necessary health care treatment and can make prescription drugs more affordable directly improving health outcomes. Individuals may have improved mental health if they are insured and less worried about their inability to meet their own health care needs in addition to the needs of their dependents.

### 4.1 Limitations

The “Healthy Days” metric, as a self‐reported measure, is intrinsically subjective. It is important to note that the results indicate shifts in perceived health; while perceived well‐being is arguably more important than diagnosed poor health, the two are not interchangeable. Healthy Days Measures appropriately map onto detailed health inventories, indicating that misreporting and discrepancies between perceived and measurable health status are likely to be minimal.^53^ Individuals may feel social pressures to report outcomes that differ from the true perception of their health—this is especially of concern when considering stigma around mental illness. We also have no reason to suspect that the accuracy of reporting would vary over time, which suggests our findings are unlikely to be an artifact of social pressures around self‐reporting. These measures are applicable outside of the U.S, further suggesting that they are valid measures of population health.^54^, ^55^ Taken collectively, the wealth of literature on this survey measure minimizes our concerns about the impact that self‐reported measures of health may have on our results.^40^

In 2011, a new sampling and weighting structure was added to BRFSS.^56^ This change incorporated the growing usage of cell phones and created less‐biased weights to more accurately reflect the underlying population. Despite these changes in methodology, there do not appear to be changes in the prevalence of our outcomes of interest following the change in weighting. Due to the nature of difference‐in‐differences analyses, modifications in weighting methodology are unlikely to differentially impact states with and without changes in EITC.^57^ Only Connecticut introduced a new refundable EITC during the time period immediately preceding or following the change in BRFSS methodology.

There could be measurement error in educational attainment used as a proxy for being most likely affected by EITC. Only the individual surveyed reported their educational attainment making it plausible for those with no more than a high school education to be residing with a college graduate. Similarly, having no postsecondary education does not mean that an individual does not have a high‐paying job and completion of college does not guarantee high wages. Nonetheless, the restriction to individuals with less educational attainment allowed us to focus on a population likely to be eligible for EITC while avoiding restrictions that might be a consequence of EITC such as income or employment. These are quasi‐experimental estimates, which could suffer from unobserved differences between states that have more and less generous EITCs. States that enact generous social policies, such as the EITC, may be more likely to have other policies that benefit low‐income households. These policies can vary from state to state, especially over the nearly three‐decade long study period. While it is unlikely that most of these policies were implemented simultaneously with EITC, they may have contributed to residual confounding.

## 5 CONCLUSIONS

During 1993‐2017, 20 states implemented refundable state Earned Income Tax Credit policies designed to increase employment and household income among lower‐earning workers. We found that these policies significantly reduced frequent mental distress and poor physical health among less‐educated adults—especially during months when the credit was most likely to be received. Interventions to reduce poverty may improve health by reducing material hardship and stress.

## CONFLICT OF INTEREST

No disclosures were reported.

## AUTHOR CONTRIBUTIONS

Ms Morgan had full access to all the data in the study and takes full responsibility for the integrity of the data and the accuracy of the data analysis.

Concept and design: Morgan, Hill, Mooney, Rivara, Rowhani‐Rahbar.

Acquisition, analysis, or interpretation of data: Morgan, Hill, Mooney, Rivara, Rowhani‐Rahbar.

Drafting of the manuscript: Morgan.

Critical revision of the manuscript for important intellectual content: Morgan, Hill, Mooney, Rivara, Rowhani‐Rahbar.

Statistical analysis: Morgan.

Administrative, technical, or material support: Morgan, Hill, Mooney, Rivara, Rowhani‐Rahbar.

Supervision: Rowhani‐Rahbar, Rivara, Hill, Mooney.

## ROLE OF THE FUNDER/SPONSOR

The sponsor had no role in the design and conduct of the study; collection, management, analysis, and interpretation of the data; preparation, review, or approval of the manuscript; and decision to submit the manuscript for publication.

## Supporting information

Supplementary Material

Appendix S1–S5

## References

[1] The World Health Organization . Poverty and Health; 2018 http://apps.who.int/iris/bitstream/10665/42690/1/9241562366.pdf. Accessed August 14, 2019.

[2] Health MP . Selection vs. Causation in the income gradient: what can we learn from graphical trends? J Health Care Poor Underserved. 2008;19(2):574‐579. 18469427 10.1353/hpu.0.0018

[3] de Ruffi T , Zdanowicz N . Effects of financial precariousness on mental health. Psychiatr Danub. 2018;30(Suppl 7):439‐442. 30439822

[4] Burns JK . Poverty, inequality and a political economy of mental health. Epidemiol Psychiatr Sci. 2015;24(2):107‐113. 25746820 10.1017/S2045796015000086 PMC6998107

[5] University of Kentucky Center for Poverty Research . UKCPR National Welfare Data, 1980–2017. http://ukcpr.org/resources/national-welfare-data. Accessed July 30, 2019

[6] Yeung W , Linver M , Brooks‐Gunn J . How money matters for young children’s development: parental investment and family processes. Child Dev. 2002;73(6):1861‐1879. 12487499 10.1111/1467-8624.t01-1-00511

[7] Currie J , Stabile M . Socioeconomic status and child health: why is the relationship stronger for older children? Am Econ Rev. 2003;93(5):1813‐1823. 29058847 10.1257/000282803322655563

[8] Galea S , Tracy M , Hoggatt KJ , DiMaggio C , Karpati A . Estimated deaths attributable to social factors in the United States. Am J Public Health. 2011;101(8):1456‐1465. 21680937 10.2105/AJPH.2010.300086 PMC3134519

[9] Glymour M , Avendano M , Kawachi I . Socioeconomic Status and Health In: Berkman L, Kawachi I, Glymour M, eds. Social Epidemiology. New York, NY: Oxford Press; 2014 17–62.

[10] Gundersen C , Ziliak JP . Childhood food insecurity in the U.S.: trends, causes, and policy options. Future Child. 2014;24(2):1‐19.

[11] Berchick ER , Barnett JC , Upton RD .Health Insurance Coverage in the United States: 2018. Washington D.C.; 2019 https://www.census.gov/content/dam/Census/library/publications/2019/demo/p60-267.pdf. Accessed July 11, 2019.

[12] Evans GW , Kantrowitz E . Socioeconomic status and health: the potential role of environmental risk exposure. Annu Rev Public Health. 2002;23(1):303‐331. 11910065 10.1146/annurev.publhealth.23.112001.112349

[13] Evans GW , English K . The environment of poverty: multiple stressor exposure, psychophysiological stress, and socioemotional adjustment. Child Dev. 2002;73(4):1238‐1248. 12146745 10.1111/1467-8624.00469

[14] Mair C , Diez Roux AV , Galea S . Are neighborhood characteristics associated with depressive symptoms? A critical review. J Epidemiol Community Heal. 2008;62(11):940‐946. 10.1136/jech.2007.066605 18775943

[15] Ross CE , Mirowsky J . Neighborhood disadvantage, disorder, and health. J Health Soc Behav. 2001;42(3):258‐276. 11668773

[16] McEwen BS . Stress and the Individual. Arch Intern Med. 1993;153(18):2093. 8379800

[17] Athanasakis K . The socioeconomic effects of uncontrolled hypertension. Curr Vasc Pharmacol. 2017;16(1):5‐9. 28412912 10.2174/1570161115666170413145125

[18] Max W . The financial impact of smoking on health‐related costs: a review of the literature. Am J Heal Promot. 2001;15(5):321‐331. 10.4278/0890-1171-15.5.321 11502013

[19] Case A , Deaton A . Mortality and morbidity in the 21st century. Brookings Pap Econ Act. 2017;2017(1):397‐476. 29033460 10.1353/eca.2017.0005 PMC5640267

[20] Internal Revenue Service . Earned Income Tax Credit. https://www.irs.gov/creditsdeductions/%0Aindividuals/earned-income-tax-credit. Accessed August 21, 2019

[21] Meyer BD . The effects of the earned income tax credit and recent reforms. Tax Policy Econ. 2010;24(1):153‐180.

[22] Center on Budget and Policy Priorities . Earned Income Tax Credit. https://www.cbpp.org/sites/default/files/atoms/files/policybasics-eitc.pdf. Accessed August 21, 2019

[23] Internal Revenue Service . EITC & Other Refundable Credits. https://www.eitc.irs.gov/eitc-central/statistics-for-tax-returns-with-eitc/statistics-for-tax-returns-with-eitc. Published 2019. Accessed September 17, 2019

[24] Strully KW , Rehkopf D , Xuan Z . Effects of prenatal poverty on infant health: State earned income tax credits and birth weight. Am Sociol Rev. 2011;75(4):534‐562. 10.1177/0003122410374086 PMC3104729 21643514

[25] Hoynes H , Miller D , Simon D . Income, the earned income tax credit, and infant health. Am Econ J Econ Policy. 2015;7(1):172‐211.

[26] Markowitz S , Komro K , Livingston M , Lenhart O , Wagenaar A . Effects of state‐level earned income tax credit laws in the U.S. on maternal health behaviors and infant health outcomes. Soc Sci Med. 2017;194:67‐75. 29073507 10.1016/j.socscimed.2017.10.016 PMC5696026

[27] Hamad R , Collin DF , Rehkopf DH . Estimating the short‐term effects of the earned income tax credit on child health. Am J Epidemiol. 2018;187(12):2633‐2641. 30188968 10.1093/aje/kwy179 PMC6269248

[28] Hamad R , Niedzwiecki MJ . The short‐term effects of the earned income tax credit on health care expenditures among US adults. Health Serv Res. 2019;54:1295‐1304. 31566732 10.1111/1475-6773.13204 PMC6863225

[29] Hamad R , Rehkopf DH . Poverty, pregnancy, and birth outcomes: a study of the earned income tax credit. Paediatr Perinat Epidemiol. 2015;29(5):444‐452. 26212041 10.1111/ppe.12211 PMC4536129

[30] Pega F , Carter K , Blakely T , Lucas P . In‐work tax credits for families and their impact on health status in adults (Review). Cochrane Database Syst Rev. 2013;6(8):CD009963. 10.1002/14651858.CD009963.pub2 PMC11649156 23921458

[31] Evans WN , Garthwaite CL . Giving Mom a break : the impact of higher EITC payments on maternal health giving mom a break : the impact of higher EITC payments on maternal health1. Am Econ J Econ Policy. 2014;6(2):258‐290.

[32] Tax Policy Center . State EITC as Percentage of the Federal EITC. https://www.taxpolicycenter.org/statistics/state-eitc-percentage-federal-eitc. Accessed July 30, 2019

[33] National Bureau of Economic Research . State EITC Provisions 1977–2018. https://users.nber.org/~taxsim/state-eitc.html. Accessed September 16, 2019

[34] Health Affairs . The Earned Income Tax Credit, Poverty, And Health. 2018 10.1377/hpb20180817.769687

[35] Meyer B . The effects of the earned income tax credit and recent reforms. Tax Policy and the Economy. 2010;24:153‐180.

[36] Grubb WN . The varied economic returns to postsecondary education: new evidence from the class of 1972. J Hum Resour. 1993;28(2):365.

[37] Heckman J , Lochner L , Todd P . Fifty Years of Mincer Earnings Regressions. Cambridge, MA: NBER Working Paper Series; 2003.

[38] Sykes J , Križ K , Edin K , Halpern‐Meekin S . Dignity and dreams. Am Sociol Rev. 2015;80(2):243‐267.

[39] Centers for Disease Control and Prevention (CDC) . Self‐reported frequent mental distress among adults–United States, 1993–1996. MMWR Morb Mortal Wkly Rep. 1998;47(16):326‐331. 9583900

[40] Moriarty DG , Zack MM , Kobau R . The centers for disease control and prevention’s healthy days measures ‐ population tracking of perceived physical and mental health over time. Health Qual Life Outcomes. 2003;1:37. 14498988 10.1186/1477-7525-1-37 PMC201011

[41] Zimmerman FJ , Anderson NW . Trends in health equity in the United States by race/ethnicity, sex, and income, 1993–2017. JAMA Netw Open. 2019;2(6):e196386. 31251377 10.1001/jamanetworkopen.2019.6386 PMC6604079

[42] Jia H . Predicting geographical variations in behavioural risk factors: an analysis of physical and mental healthy days. J Epidemiol Community Heal. 2004;58(2):150‐155. 10.1136/jech.58.2.150 PMC1732662 14729899

[43] Dwyer‐Lindgren L , Mackenbach JP , van Lenthe FJ , Mokdad AH . Self‐reported general health, physical distress, mental distress, and activity limitation by US county, 1995–2012. Popul Health Metr. 2017;15(1):16. 10.1186/s12963-017-0133-5 PMC5406923 28446196

[44] Cordier T , Slabaugh SL , Havens E , et al. A health plan’s investigation of healthy days and chronic conditions. Am J Manag Care. 2017;23(10):e323–e330. 29087635

[45] Casebeer AW , Antol DD , Hopson S , et al. Using the healthy days measure to assess factors associated with poor health‐related quality of life for patients with metastatic breast, lung, or colorectal cancer enrolled in a Medicare advantage health plan. Popul Health Manag. 2019;22(5):440‐448. 31211653 10.1089/pop.2019.0054

[46] Kaiser HJ , Foundation KFF .State Health Facts. https://www.kff.org/other/state-indicator/smha-expenditures/?currentTimeframe=0&sortModel=%7B%22colId%22:%22Location%22,%22sort%22:%22asc%22%7D. Accessed September 17, 2019.

[47] Wing C , Simon K , Bello‐Gomez R . Designing difference in difference studies: best practices for public health policy research. Annu Rev Public Health. 2018;39:453‐469. 29328877 10.1146/annurev-publhealth-040617-013507

[48] Rehkopf DH , Strully KW , Dow WH . The short‐term impacts of earned income tax credit disbursement on health. Int J Epidemiol. 2014;43(6):1884‐1894. 25172139 10.1093/ije/dyu172 PMC4342690

[49] Lenhart O . The effects of state‐level earned income tax credits on suicides. Health Econ. 2019;28:1476‐1482. 31469485 10.1002/hec.3948

[50] Dow WH , Godøy A , Lowenstein CA , Reich M , Way C . Can Economic Policies Reduce Deaths of Despair? 2019 http://www.nber.org/papers/w25787. Accessed August 20, 2019.

[51] Dieleman JL , Baral R , Birger M , et al. US spending on personal health care and public health, 1996–2013. JAMA. 2016;316(24):2627. 28027366 10.1001/jama.2016.16885 PMC5551483

[52] Platt S . Unemployment and suicidal behaviour: a review of the literature. Soc Sci Med. 1984;19(2):93‐115. 6382623 10.1016/0277-9536(84)90276-4

[53] Hennessy CH , Moriarty DG , Zack MM , Scherr PA , Brackbill R . Measuring health‐related quality of life for public health surveillance. Public Health Rep. 109(5), 665‐672. 7938388 PMC1403555

[54] Centers for Disease Control and Prevention (CDC) . Health‐related quality of life–Puerto Rico, 1996–2000. MMWR Morb Mortal Wkly Rep. 2002;51(8):166‐168. 11900118

[55] Ounpuu S , Krueger P , Vermeulen M , Chambers L . Using the U.S. Behavior Risk Factor Surveillance System’s health related quality of life survey tool in a Canadian city. Can J Public Health. 91(1), 67‐72. 10765590 10.1007/BF03404258 PMC6980029

[56] CDC . Behavioral Risk Factor Surveillance System. 2017 https://www.cdc.gov/brfss/annual_data/2016/pdf/compare_2016.pdf. Accessed April 24, 2019.

[57] Geiss LS , Kirtland K , Lin J , et al. Changes in diagnosed diabetes, obesity, and physical inactivity prevalence in US counties, 2004–2012. Kaser S, ed. PLoS One. 2017;12(3);e0173428. 28267760 10.1371/journal.pone.0173428 PMC5340361

