# Supplementary material for: State earned income tax credits and general health indicators: A quasi‐experimental national study 1993‐2016
Source: Health Serv Res. 2020 Jul 8;55(Suppl 2):863–72. doi: 10.1111/1475-6773.13307 (PMC7518814; doi:10.1111/1475-6773.13307)
Supplement: Supplementary file 2 — Appendix S1–S5 [file HESR-55-863-s002.docx]

**Appendix**

**State Earned Income Tax Credits and General Health Indicators:**

**A Quasi-Experimental National Study 1993–2016**

**APPENDIX S1: BRFSS Respondent Characteristics Among Respondents Without Postsecondary Education by State EITC Status**

|  | No EITC during study period | EITC for full study period | Introduction of EITC during study period |
| --- | --- | --- | --- |
| Female | 52.4% (52.3, 52.6) | 50.8% (50.4, 51.2) | 52.1% (51.9, 51.2) |
| Children <18 in Household | 39.6% (39.5, 39.8) | 34.3% (33.8, 34.7) | 41.9% (41.7, 42.1) |
| Race/Ethnicity |  |  |  |
| White | 69.9% (69.7, 70.1) | 88.8% (88.6, 89.1) | 61.6% (61.3, 61.8) |
| Black | 12.8% (12.7, 12.9) | 4.0% (3.8, 4.2) | 11.5% (11.4, 11.6) |
| Asian | 1.1% (1.1, 1.1) | 1.1% (1.0, 1.2) | 2.0% (2.0, 2.1) |
| AIAN | 1.1% (1.0, 1.1) | 1.3% (1.2, 1.4) | 1.2% (1.2, 1.3) |
| Hispanic | 13.7% (13.6, 13.9) | 3.8% (3.6, 4.0) | 22.2% (22.0, 22.4) |
| Other | 1.5% (1.4, 1.5) | 1.0% (0.9, 1.1) | 1.5% (1.5, 1.5) |
| Marital Status |  |  |  |
| Married | 55.0% (54.9, 55.2) | 55.8% (55.4, 56.2) | 52.6% (52.4, 52.7) |
| Divorced/Separated | 13.0% (12.9, 13.1) | 10.4% (10.5, 10.6) | 12.4% (12.3, 12.5) |
| Widowed | 9.9% (9.8, 10.0) | 9.9% (9.7, 10.1) | 9.6% (9.5, 9.7) |
| Never Married | 22.1% (21.9, 22.2) | 23.9% (23.5, 24.3) | 25.5% (25.3, 25.7) |
| **Abbreviations:** EITC: Earned Income Tax Credit; AIAN: American Indian Alaska Native  **Notes:**  **-** Above proportions represent demographics of respondents during entire study period  - Proportions/responses are survey-weighted to be generally representative of the underlying population  - Race/ethnicity has been bridged to accommodate multiple iterations of the questionnaire and may not perfectly reflect respondent identity | | | |

**APPENDIX S2: Selected Behavioral Risk Factor Surveillance System Questions**

**Would you say that in general your health is—**

1 Excellent

2 Very good

3 Good

4 Fair

5 Poor

7 Don’t know / Not sure

9 Refused

**Now thinking about your mental health, which includes stress, depression, and problems with emotions, for how many days during the past 30 days was your mental health not good?**

_ _ Number of days [RANGE = 1-30]

8 8 None

7 7 Don’t know / Not sure

9 9 Refused

**Now thinking about your physical health, which includes physical illness and injury, for how many days during the past 30 days was your physical health not good?**

_ _ Number of days [RANGE = 1-30]

8 8 None

7 7 Don’t know / Not sure

9 9 Refused

**APPENDIX S3: Visual Assessment of Parallel Trends Assumption**

C.1 Prevalence of suboptimal overall health prior to EITC implementation


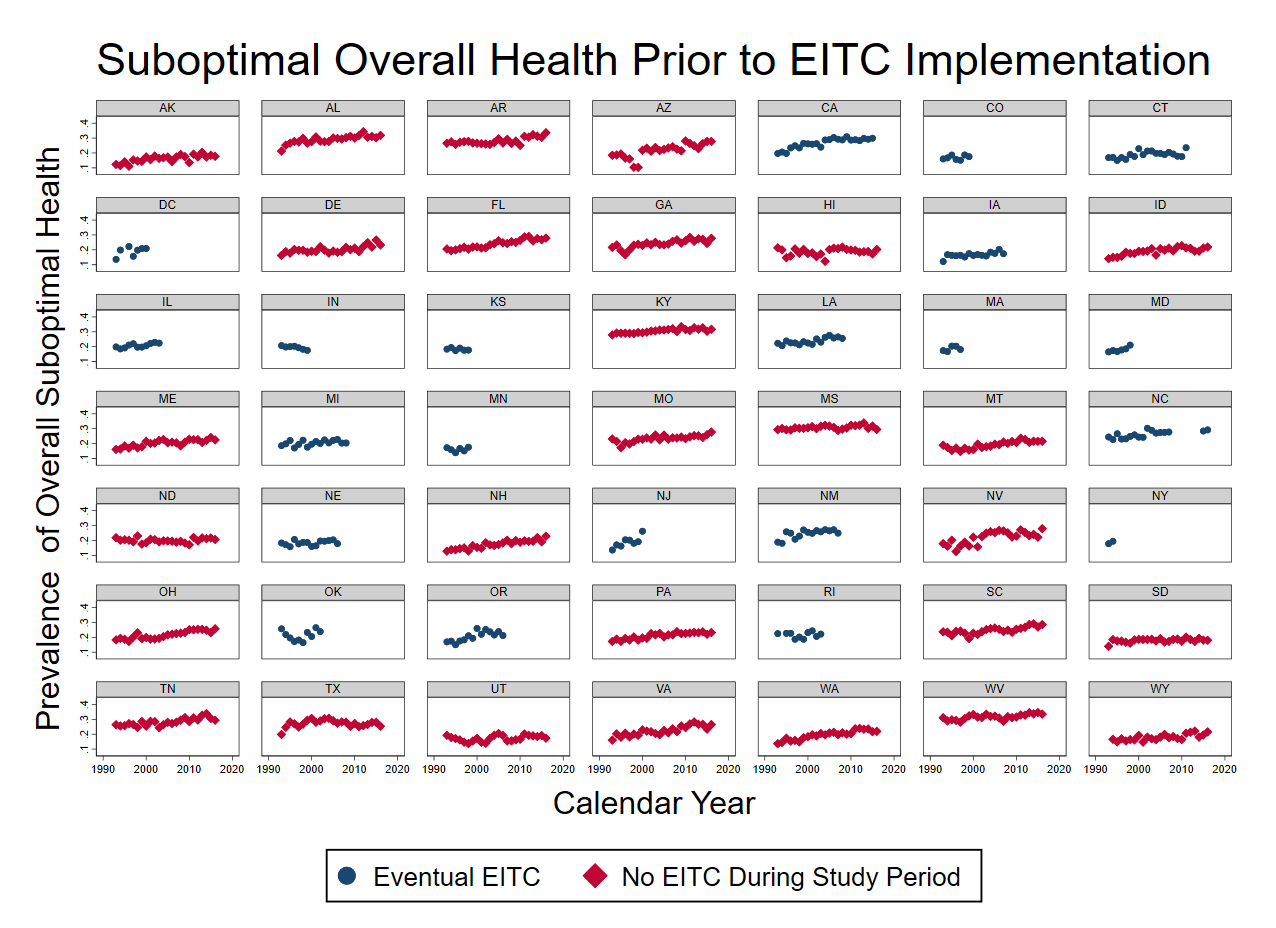


C.2 Prevalence of FMD prior to EITC implementation

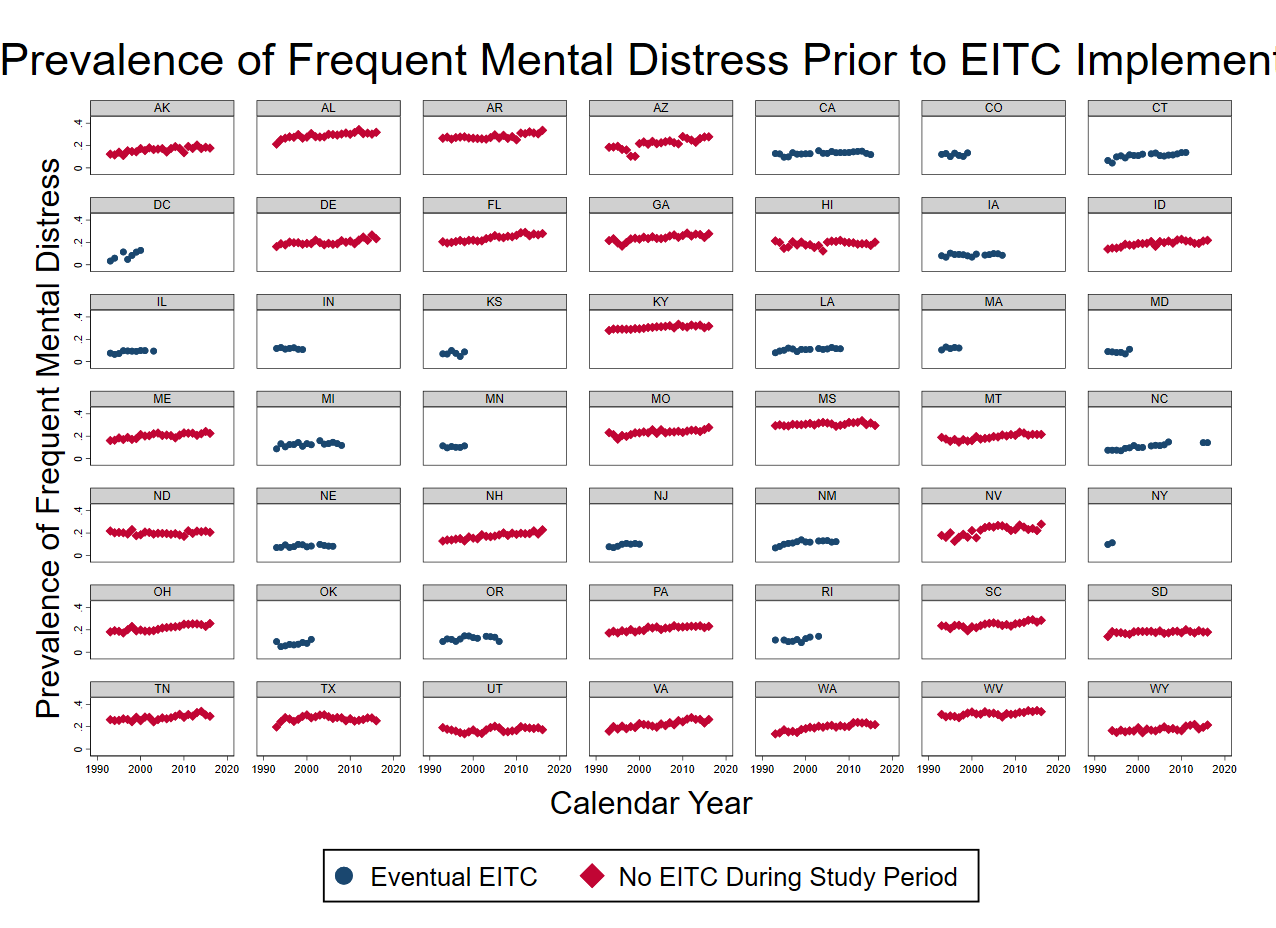


C.3 Prevalence of FPPH prior to EITC implementation
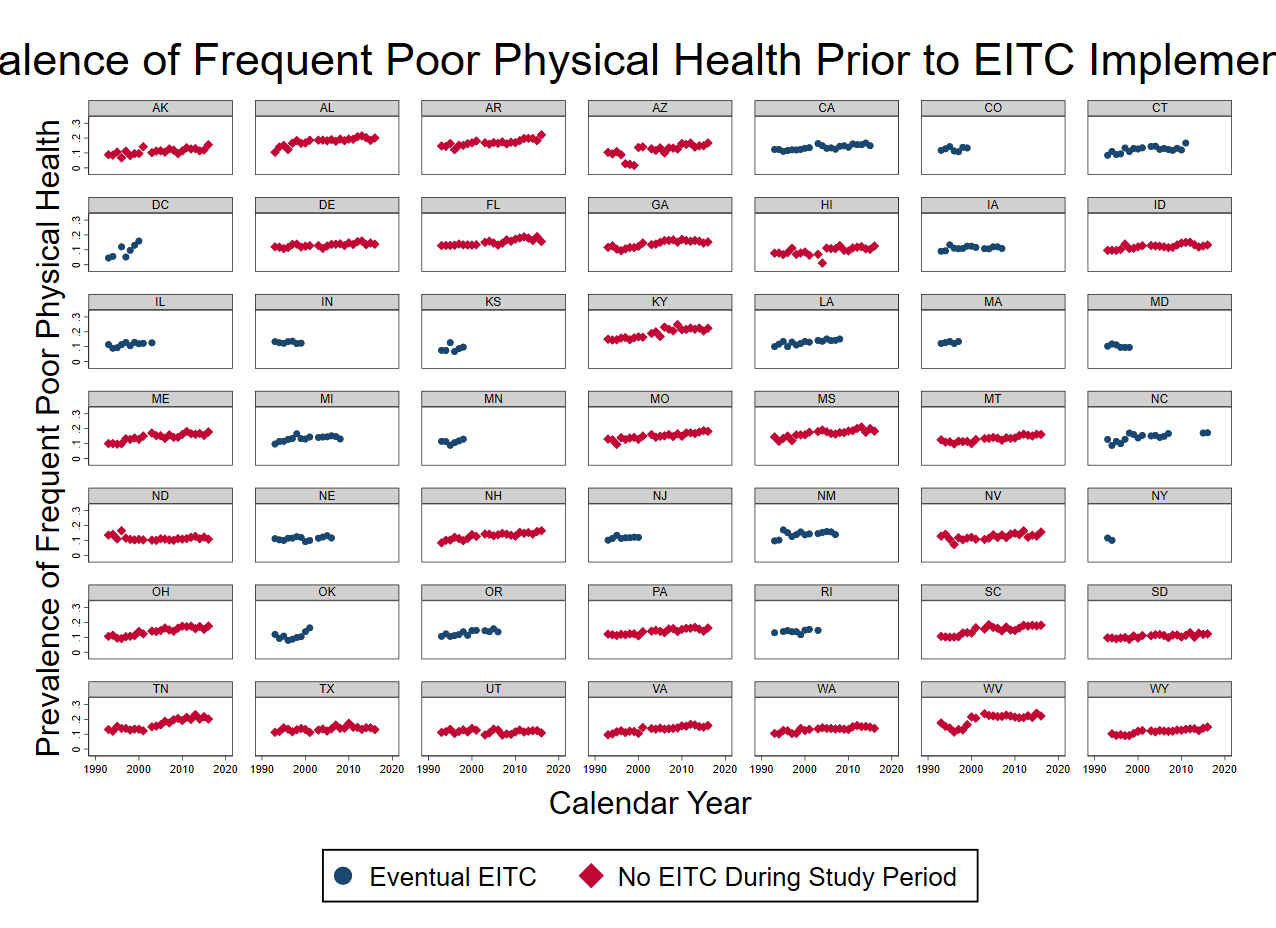


**APPENDIX S4: Differences in Prevalence of Reported Poor Health—Calculated with Weighted Linear Regression**

| **Differences in Prevalence of Poor Health Reported on BRFSS per 100,000 Population with Maximum Educational Attainment of High School Diploma—Calculated with Weighted Linear Regression, 1993–2016** | | |
| --- | --- | --- |
|  | Crude PD | Adjusted PD**†** |
| Overall Suboptimal Health | 27.5 (-393.6, 448.5) | 0.6 (-556.9, 558.1) |
| FMD | *-288.1 (-493.0, -83.2)* | -162.4 (-382.1, 57.4) |
| FPPH | *-241.9 (-436.4, -47.4)* | *-197.1 (-390.6, -35.5)* |
| Models calculated using linear regression. Observations are state-year prevalence of outcome among those with no post-secondary education. Each state-year point is weighted by size of population without a college degree.  **†**Adjusted for state GDP, state minimum wage, and adoption of Medicaid expansion  BRFSS: Behavioral Risk Factor Surveillance System, PD: Prevalence Difference, FMD: Frequent Mental Distress, FPPH: Frequent Poor Physical Health | | |

**APPENDIX S5: Differences in Prevalence of Reported Poor Health Among Female Respondents**

| **Risk Differences For 10 Percentage Point Increase in EITC Among Female† BRFSS Respondents** | | | | |
| --- | --- | --- | --- | --- |
|  | Year-Round | | February, March, April | |
|  | Crude PD | Adjusted PD**‡** | Crude PD | Adjusted PD**‡** |
| Overall (Suboptimal) | 76.3 (-104.3, 256.9) | 90.3 (-98.5, 279.1) | 55.1 (-334.8, 444.9) | 89.6 (-318.6, 497.8) |
| Mental | *-401.3 (-574.5, -228.0)* | *-219.0 (-404.1, -34.0)* | *-655.2 (-1023.3, -287.1)* | *-515.6 (-905.4, -125.8)* |
| Physical | *-317.8 (-481.5, -154.1)* | *-249.8 (-422.1, -77.6)* | *-368.3 (-732.0, -4.5)* | -318.7 (-697.0, 59.6) |
| **†** Historically, BRFSS interviewers have been instructed to guess the sex of the respondent based on the sound of their voice. While some states have more recently encouraged the interviewers to ask and even include modules assessing sex and gender independently, this is not consistent between or within state-years.  **‡** Adjusted for state GDP, state minimum wage, and adoption of Medicaid expansion  BRFSS: Behavioral Risk Factor Surveillance System, PD: Prevalence Difference, FMD: Frequent Mental Distress, FPPH: Frequent Poor Physical Health | | | | |
